# Development and validation of the Scale of Motives for Using Social Networking Sites (SMU-SNS) for adolescents and youths

**DOI:** 10.1371/journal.pone.0225781

**Published:** 2019-12-03

**Authors:** Miguel-Ángel Pertegal, Alfredo Oliva, Ana Rodríguez-Meirinhos

**Affiliations:** 1 Departamento de Psicología Evolutiva y de la Educación, Universidad de Sevilla, Seville, Spain; 2 Department of Communication and Education, Universidad Loyola Andalucía, Seville, Spain; Aalborg University, DENMARK

## Abstract

Over the past decade, the Uses and Gratifications theory has driven research on the motives behind social media use. The three most commonly explored motives have been: maintaining relationships, seeking information, and entertainment. The aim of this study was to develop and validate the Scale of Motives for Using Social Networking Sites (SMU-SNS), a measure to assess a wider range of motives for using Social Networking Sites than have previously been researched. A multi-method design with different samples of high-school and university students was used. First, to develop the pool of items, a literature review and a focus group study (*n* = 48, age range = 16–21) was conducted. Second, to reduce and refine the pool of items a pilot study (*n* = 168, age range = 14–24) was performed. Third, a validation study (*n* = 1102, age range = 13–25) was conducted to assess the validity and reliability of the SMU-SNS. Cross-validation using EFA and CFA resulted in a final version comprising 27 items distributed in nine factors (Dating, New Friendships, Academic Purposes, Social Connectedness, Following and Monitoring Others, Entertainment, seeking Social Recognition, Self-expression, and seeking Information). Internal consistency was excellent and evidence of measurement invariance across gender and age was largely achieved. The SMU-SNS scores significantly correlated with other relevant variables, including age, gender, certain personality traits, social support, loneliness, and life satisfaction. Overall, findings supported the SMU-SNS as a valid and reliable measure to assess youth’s motives for using Social Networking Sites. Psychometric and general implications are discussed.

## Introduction

### The current state of research on motives for using Social Networking Sites (SNS)

The widespread use of Social Networking Sites (SNS), especially among adolescents and youth, has piqued the interest of researchers to study the motives behind this use, resulting in a number of recent publications highlighting the potential impact of SNS and its motives on users’ psychological and social wellbeing. Previous studies on motivations for using SNS [1] indicate that each used scale differs significantly from each other in the motives they explore. Thus, we consider necessary to achieve some consensus or classification of the relevant motives to be researched. Besides, many studies have focused on describing the motives of use, without looking into the relationships between these motives and some users’ psychosocial development outcomes. Indeed, a few recent studies have evidenced the importance of the motives for using SNS by showing its relationship not only with SNS addiction [2], but also with the subjective and social users’ wellbeing [3,4]. However, the lack of agreement about which motives should be explored hinders the comparison between studies and, thus, the possibility to achieve clear conclusions.

For this reason, it seems necessary to develop a valid and reliable scale that includes a broad range of motives for using SNS. Few studies have employed a validated scale that allows a wide and significant range of motives for using SNS to be explored using rigorous quality criteria. This article presents a new scale purposely designed to measure adolescents and youths’ motives, developed as a tool for professionals interested in studying SNS and the motives of use.

The range of motives for using SNS is wide, from personal or private use, to professional and commercial use. Because of this, there are several models from the Social Commerce trend that have been developed to understand the factors that explain the intention to use, that is, the intention of continuous consumption of SNS through time, proposing several constructs such as purchase intention, word of mouth, and so on [5,6]. However, the present study is focused on the motives that explain the experience of the personal/private use of SNS from a behavioural and psychological approach.

The Uses and Gratifications Theory [7–9] has commonly been used as a framework for explaining the motives for using the Internet as well as SNS. The fundamentals of this theory are as follows: people use an application or social network because they anticipate certain gratifications; the user finds at least partial gratification after its use, for example, to pass time or find information; this gratification reinforces future use. Many studies have been derived from this theory that try to explain the intentions behind the use of SNS, introducing the users’ motivations as key variables in these explicative models.

In order to pinpoint the variety of motives for using SNS, a literature review was conducted. The first studies related to motives of use were fundamentally descriptive and focused on examining SNS users’ main motivations [10–13]. For instance, Brandtzaeg & Heim [10], after an exhaustive qualitative analysis, proposed 12 motives in descending order of frequency: seeking new relations, keeping in touch with friends and acquaintances, general socializing, accessing information, debating, free short messaging service, time-killing, sharing/consuming content, unspecified fun, profile surfing, keeping in touch with family, and others (e.g., promoting one’s own work).

Although there is a general lack of a rigorous systematic review regarding the reasons for using SNS, some relevant attempts to classify the identified motives have been made more recently. Such is the case of Krasnova, Veltri, Eling, & Buxmann [14], concluding in their literature review that there are four fundamental types of motives: relationship building, self-enhancement, informational benefits, and entertainment seeking. It is an interesting, albeit incomplete, classification of the principal types of motives.

Given the absence of a systematic review and a rigorous classification of the types of identified motives of use, in a previous study we conducted a review of more than one-hundred published works about the experience of SNS use. In this study we built a taxonomy of nine main themes about this experience. One of these themes was related to motives of use that included a synthesis of seven principal types of motives of use studied to-date (Table 1). Detailed information concerning this review is provided in a previously published paper [1].

**Table 1:** Main types of motives for using SNS studied to date.

1. Seeking information motives
1.1. Access to general information (*n* = 48)
1.2. Monitoring Others (*n* = 17)
1.3. Academic purposes (*n* = 13)
1.4. Usefulness and gaining information (*n* = 7)
2. Motives related to positive sensations:
2.1. Entertainment (*n* = 60)
2.2. Passing time (*n* = 28)
2.3. Escape/Escapism (*n* = 14)
2.4. Flow (*n* = 3)
3. Maintaining relationships:
3.1. Keep in touch with close friends (*n* = 46)
3.2. Keep in touch with other friends (old or distant friends, acquaintances, etc.) (*n* = 43)
3.3. Maintaining latent ties (*n* = 3)
3.4. Keep in touch with family (*n* = 3)
4. Motives related to establishing new relationships:
4.1. Making new friends (*n* = 24)
4.2. Dating purposes (*n* = 7)
4.3. Camaraderie (*n* = 4)
4.4. Adding more friends (*n* = 3)
5. Social recognition motives:
5.1. Seeking social recognition and social enhancement (*n* = 24)
5.2. Coolness (*n* = 18)
5.3. Convenience (*n* = 10)
5.4. Stylishness (*n* = 2)
6. Social connectedness motives:
6.1. Virtual community (*n* = 12)
6.2. Social identity (*n* = 4)
6.3. Social inclusion (*n* = 4)
6.4. Group identity (*n* = 2)
7. Self-expression motives:
7.1. Emotional self-expression (*n* = 17)
7.2. Expression of opinions (*n* = 13)
7.3. Self-discovery (*n* = 2)
7.4. ‘Testing ground’ for social behavior (*n* = 2)

The table above shows that entertainment, seeking information, and keeping in touch were the principal motives for SNS use in the literature reviewed. The aforementioned seven categories are broad, that is to say, they are motive types that simultaneously include other specific motives that may be worth differentiating for research. Such is the case in the category of establishing new relations, which may include the motivation for making new friends to looking to date or meet partners through SNS which we feel should be studied separately. Therefore, a scale that aims to evaluate motives of use must not only consider the aforementioned seven principal types, but also tap into certain specific motives of use.

### Gender and age differences in motives for SNS use

A considerable number of studies have analyzed gender differences in the context of SNS use [11–31]. These studies have convincingly indicated that girls more frequently use SNS to follow and keep in touch with existing friends, whereas boys report using them more to establish new relationships and even to establish romantic relationship. There is also evidence that girls–compared to boys–use SNS more frequently for entertainment, social recognition, or access to social information. With regards to other types of motives, there are either no differences or the results are contradictory, keeping us from drawing clear conclusions.

Although gender and age may influence the motives for using SNS, the cited studies have not explicitly tested the measurement invariance of the scale employed. Therefore, these conclusions should be confirmed using a scale with evidence that the constructs being measured are similar across groups (i.e., boys and girls) to determine whether the reported differences are real or simply the result of a methodological artifact (i.e., differences in the structure of the scale or the meaning of the items)

The few studies analyzing the relationship between motives of use and age have hardly found differences [11,21,28,30–33]. Most of them did not include samples covering the entire range of ages from adolescence to early adulthood and therefore do not inform about the developmental trends in the motives for using SNS. Nonetheless, the available data shows two tendencies. On one hand, older users report using SNS for motives related to maintaining contact with friends, whereas younger users use SNS more to establish new friendships and relationships. In addition, for the youngest users SNS are more important for obtaining social recognition or to feel included in an online community. Lastly, whereas the youngest users generally use SNS more for entertainment, the older users report to use them more to stay informed or for academic purposes, amongst other motives.

### Psychological variables and motives of SNS use

The hypothetical relationship between motives of use and certain psychological variables can be approached from two points of view. Thus, whereas certain traits or personality dimensions can be considered as antecedents (i.e., determinants of SNS motives of use), other variables such as individuals’ wellbeing or psychological maladjustment could be a consequence of how SNS is used.

Firstly, it should be noted that studies have traditionally focused on the relationship between certain personality traits and the frequency of SNS use [34,35]. Specifically, extraversion and neuroticism are especially associated with SNS use. The available data shows a positive association between extraversion and the motives related to establishing new relationships [22,28,31,36], seeking social recognition [36,37], and entertainment in the sense of enjoyment and leisure through SNS [28,31]. On the other hand, neuroticism has been positively associated with surveillance and passive following of other users [38,39], the search for general information [38,39], seeking approval from others [31,37], or using SNS as a way of escape [31]. On the contrary, neuroticism has been negatively associated with establishing new relationships [36]. Regarding other personality factors such as consciousness or agreeableness, amongst others, there are few clear and conclusive results.

The relationship between motives of use and other variables such as life satisfaction or perceived social support have been analyzed even less. The motives related to maintaining contact and following friends has been positively associated with higher perceived social support in real life and therefore with lower perceived loneliness [40]. Based on the same logic, this type of motive does not only associate with social wellbeing but is also related to higher perceived life-satisfaction [22,41,42].

### The present study

The present study aimed to develop and validate a scale to assess the motives of adolescents and youth for using SNS. Given that instruments to assess the motives for SNS-use in this age group are still scarce and the measures available are specific to certain social networks, we will adopt an exploratory approach (i.e., literature review and qualitative focus groups) to determine the dimensions underlying the construct of motives for SNS use. Valid evidence based on the internal structure (i.e., dimensionality, internal consistence, and measurement invariance across gender and age) and relationships with other variables will be provided. Gender and age-based differences in the motives for using SNS will also be researched.

## Materials and methods

Approval to conduct this study was obtained in written form by the Biomedical Research Ethics Review Board of Andalucia-Spain (C.P. PSI2015-64211-R—C.I. 0462-N-16).

### Sample and procedure

The sample was comprised of 1102 adolescents and youth. Out of the total sample surveyed, 94.28% reported using SNS and filled out the questionnaires. Thus, the final sample included 455 boys and 584 girls between 13 and 25 years old (*M* = 16.89, *SD* = 2.50). Most participants were born in Spain (95.9%) and all of them were Spanish-speaking, so they filled in the questionnaires in Spanish. The distribution of participants according to the age groups of the full sample was: 34% between 13 and 15 years old, 43.2% between 16 and 18 years old, and 22.8% above 18 years old. They were recruited from 9 high-schools and 3 university faculties (education, biology, and architecture) located in the Spanish region of Western Andalusia. The high schools were selected using a quota sampling taken according to the size of municipality (< 30.000 inhabitants for small municipalities and ≥ 30.000 inhabitants for large municipalities) and ownership (public or private). Faculties were chosen from three different areas of knowledge: social sciences, health sciences and technical sciences. Distribution of participants according to their educational status was as follows: secondary education (37.60%), post-secondary education (30.30%), professional training (11.2%), and university (20.9%). Size of the sample was determined according to Roquette and Falissard [43] who established a minimum requirement of 300 observations for the internal validation of scales. All participants or their parents if they were under 18, provided written informed consent prior to participation. They filled out the questionnaires during ordinary class periods in the presence of a research assistant.

### Measures

#### Scale of Motives for Using Social Networking Sites (SMU-SNS)

The process of development and validation of the measure to assess motives for using SNS was conducted and described according to the sequential order specified in the guidelines by MacKenzie, Podsakoff, & Podsakoff [44].

Three methods were used to develop the conceptual definition of the measured construct: a comprehensive literature review, a review of existing measurement tools, and a focus groups study. Firstly, a comprehensive literature review about the motives for using Social Networking Sites (SNS), which served to define the purpose of the scale and the dimensions to be assessed [1].

In a second step, existing measurement tools were reviewed to identify which motives were generally included and to detect gaps in their coverage. In order to design a scale that covers the entire range of possible motives for using SNS specified in Table 1, we conducted a detailed review of the existing instruments. Table 2 reports the identified scales that include at least two dimensions and whose structure was explored using factorial analysis.

**Table 2:** Description of the previous studies developing scales to assess motives for using SNS.

	Descriptives (*N; M*age / population; Country; SNS)	Data analyses			Motives													
		EFA	CFA	External validity	1	2	3	4	5	6	7	8	9	10	11	12	13	14
Aladwani [45]	416; 20.13±1.42; Kuwait; Facebook	✔	✔		✔		✔		✔	✔		✔					✔	✔
Cheung, Chiu, & Lee [46]	182; University students; China; Facebook	✔	✔		✔		✔			✔		✔			✔			✔
Dhir et al. [47]	579; High school and college students; India; Facebook		✔		✔	✔	✔						✔			✔		✔
Gan & Li [48]	297; University students; China; WeChat	✔	✔		✔		✔			✔					✔			✔
Giannakos, Chorianopoulos, Giotopoulos, & Vlamos, [15]	222; 95% Youngers; Greece; Facebook	✔	✔												✔			✔
Ha et al. [7]	361; wide range of age (18 to 59); South Korea; Facebook	✔			✔		✔					✔						✔
Horzum [37]	621; 21.27±1.76; Turkey; Facebook	✔	✔	✔	✔	✔	✔					✔	✔			✔		✔
Hughes, Rowe, Batey, & Lee [38]	300; 27±8.98; International(online); Twitter & Facebook	✔		✔	✔							✔						
Hunt, Atkin, & Krishnan [16]	417; 18.9±.95; EEUU; Facebook				✔		✔		✔			✔						
Infinedo [49]	797; University students; 4 American countries: EEUU, Canada, Mexico & Argentina		✔		✔		✔				✔		✔			✔		
Krasnova et al. [14]	448; 25.8±5.7; Germany; SNS	✔			✔					✔	✔		✔				✔	
Ku, Chu, & Tseng [50]	122; University students; Taiwan; SNS	✔			✔		✔				✔		✔			✔		
Lien & Cao [51]	264; Youngers (18 to 30); EEUU; Facebook	✔			✔		✔											
Sheldon [13]	172; 19.93±1.23; EEUU; Facebook	✔														✔	✔	✔
Sheldon & Bryant [52]	253; 20±2.94; EEUU; Instagram	✔		✔			✔					✔			✔			
Tosun [53]	143; 22.63±3.63 Turkey; Facebook	✔																
Xu, Ryan, Prybutok, & Wen [54]	148; University students; EEUU; SNS	✔		✔			✔					✔			✔	✔		✔
Zhang, Li, Wu, & Li [55]	240; Wide range of age (18 to >60); China; WeChat	✔			✔		✔			✔								✔

Note: 1 = Information; 2 = Academic purposes; 3 = Seeking positive sensations; 4 = Expression of emotions; 5 = Expression of opinions; 6 = Social recognition and self-enhancement; 7 = Keep in touch with close friends; 8 = Maintaining relationships; 9 = Making new Friends; 10 = Dating purposes; 11 = Social connectedness; 12 = Coolness and influence; 13 = Following and monitoring others; 14 = Others.

It should be noted that only some publications detailed the validation process or offered evidence on different types of validity [37, 45–46]. A large majority of studies only conducted an Exploratory Factor Analysis (EFA), while less than half of them cross-validated the scale structure through Confirmatory Factor Analysis (CFA). Moreover, it was exceptional to provide evidence of external validity with psychosocial variables, with some studies exploring the relationships between motives and other use of SNS variables through a Structural Equation Model (SEM). As can also be observed in Table 2, no scale explored all of the motives that we intended to research in our study, which motivated us to build a scale to this end.

Additionally, in a third step, we conducted 6 focus groups with 48 high-school and university students ranging in age from 16 to 21 (50% girls) to explore their motives for using SNS. In order to initiate the group discussion, participants were asked to discuss two open-ended questions: “What do you think people use SNS for?” and “What are the motives that lead you to use SNS?”. Afterwards, they were asked more specific questions with the objective of researching more specific participants´ motives for using SNS. Focus groups were recorded and transcribed verbatim for analysis and coding using the NVIVO11 program. Data were open-coded by two authors separately who looked for key motives for using SNS. Reduction was achieved by examining commonality or similarity in meaning. Differences were discussed with the research team and resolved by consensus. Transcripts were reviewed at least 5 times. Ten motives for using SNS were identified (Table 3), however only the most frequently mentioned motives (*n* > 10) were included in the scale.

**Table 3:** Themes from focus groups and frequency of appearance.

Motives for using SNS	Examples	# of times mentioned
Entertainment	“I use it out of boredom”, “For those little moments in which you don’t know what to do”, “I entertain myself watching short clips”, “It’s a way of distracting myself all day long”	37
Popularity	“There’s too many people that needs likes”, “Some people expose themselves too much just to receive likes”, “If I have many likes, it uplifts my mood”	27
Keeping in touch	“I use it only to keep in touch with the people I know”, “To know the whereabouts of friends I haven’t seen for some time”, “To tell your friends “Come on, let’s go out””	20
Information	“To see what has happened today”, “It’s a way of getting information without watching TV or reading a newspaper”, “I get to know about a lot of things”	16
Coolness	“It’s the trend, everybody uploads things”, “It’s cool now to upload streamings and stories”, “I enjoy following fashionable people”	16
Making new friends	“I hope that the person I’m interested in will like my posts several times and will end up writing to me”, “Some accept friendship requests more easily to connect with new people”	12
Curiosity and following others	“To know the whereabouts of everybody, that’s the first motive”, “If I stay away of SNS I won’t know if my friends are going on a trip or if they are studying or if they’ve passed”	11
Convenience and group influence	“The world and your peers force you”, “You want to be where your friends are, where everyone is”	5
Sharing moments	“If you don’t post that you went out, it’s like you didn’t”, “People publish everything, whatever happens to them, everything”	3
Social inclusion	“Nowadays, if you don’t have SNS, it’s like you were not a complete person”, “You have to be, if not… you are lost from people, out of everything”, “If you shut down your SNS, you will have to come back, because it would be like a social suicide”	2

Based on the aforementioned methods, 13 dimensions were finally identified by the research group as relevant, including: Dating, New Friendships, Keeping in Touch with Close Friends, Keeping in Touch with Old or Distant Friends, Academic Purposes, Social Connectedness, Following and Monitoring Others, Entertainment, Coolness, Influencer, Self-expression, Information, and Professional Purposes.

With regard to the development of the measure and model specification, and in order to represent these constructs, we generated an initial pool of 84 items to represent the measured constructs. These items then underwent a revision process for the sake of representativeness, comprehensiveness, simplicity, and clarity. The content validity of the initial pool was assessed by an expert who evaluated the item’s relevance in relation to the construct. This process resulted in 65 items (5 items per dimension) to be answered in a 7-point Likert scale from 1 (*completely untrue*) to 7 (*completely true*), indicating if they used the SNS for given reasons.

To evaluate and refine the scale, the initial 65-item version of the SMU-SNS was pretested on a sample of 168 high-school students or first-year university students of education with a mean age of 17.03 (*SD* = 2.57; range = 14 to 24 years-old), and who all reported to use SNS. The purpose of this stage was to collect data that allows to refine and reduce the initial pool of items by conducting several preliminary item-level analyses. In order to retain maximally informative and distinct items, four overly-similar items and seven items with wording considered ambiguous, too long, or that may cause confusion among respondents were removed. Three additional items with inter-item correlation above .70 and eight items with poor corrected item-total or inter-item correlations (below .40) were eliminated. Furthermore, five items were discarded because of skew or kurtotic distributions. A floor-effect regarding the five items of professional motives indicated that participants, most likely because they were students, did not use SNS for these purposes. Therefore, this dimension was eliminated from the scale, as it did not appear to address the youth’s motivations for using SNS. In addition, the distinction between old or distant friends versus close friends was confusing for participants. After the refinement process, there were not enough remaining items to constitute two independent dimensions and therefore the items were merged into a single dimension labeled Keeping Touch with Friends. In summary, 32 items were removed during this process, yielding a reduced pool of 33 items addressing eleven dimensions.

Information of the validation phase is provided on the results section.

#### Big Five Inventory-10 (BFI-10)

Extraversion and neuroticism personality traits were assessed with the Big Five Inventory-10 [56], which demonstrated satisfactory test-retest reliability and convergent validity with other personality measures [57]. The BFI-10 contains 10 items that evaluate five personality traits (extraversion, agreeableness, conscientiousness, neuroticism, and openness). However, only the extraversion and neuroticism subscales were used in this study. The prompt “I see myself as someone who…” was followed by four items that measured the personality dimensions of extraversion (e.g., “is outgoing, sociable”), and neuroticism (e.g., “get nervous easily”) in a 5-point Likert scale from 1 (*strongly disagree)* to 5 (*strongly agree*). Cronbach alphas in the present study were .69 for extraversion and .75 for neuroticism.

#### Satisfaction with Life Scale (SWLS)

The cognitive aspect of subjective wellbeing was assessed using the Satisfaction with Life Scale [58]. The scale includes 5 items rated on 7-point Likert scale ranging from 1 (*strongly disagree*) to 7 (*strongly agree*) and provides a global cognitive judgment of one’s life satisfaction. A sample item reads: “In most ways, my life is close to my ideal”. Cronbach’s alpha was .80.

#### Multidimensional Scale of Perceived Social Support (MSPSS)

The Multidimensional Scale of Perceived Social Support [59] is a 12-item self-report measure designed to assess the respondent’s perception of available social support from three sources: family, peers, and significant other. In this study, only the four-item peer subscale was used. Items (e.g., “I can talk with someone about my problems”) were rated on a 7-point Likert scale ranging from 1 (*strongly disagree*) to 7 (*strongly agree*). Cronbach’s alpha was .93.

#### UCLA-8 Loneliness Scale

A short-form measure (UCLA-8) [60] of the UCLA Loneliness Scale (UCLA-20) [61] was administered. The UCLA-8 measures subjective feelings of loneliness and social isolation through 8 items answered on a 4-point Likert scale ranging from 1 (*never*) to 4 (*very often*). A sample item reads: “I lack companionship”. Cronbach’s alpha was .88.

### Data analysis

The study of the validity of the final 33-item version of the SMU-SNS was conducted in several stages. Firstly, a number of item-level analyses were conducted. In a second stage, the dimensionality was examined through a cross-validation process using Exploratory Factor Analysis (EFA) and Confirmatory Factor Analysis (CFA). Following Osborne [62] recommendations, the sample was divided into two split-half random samples (*n*_1_ = 517, *n*_2_ = 522), preserving the relative distribution of participants according to gender, *χ*^2^(1) = .04, *p* > .05, and age, *t*(1037) = .44, *p* > .05.

The first half-split sample was used to conduct a series of EFAs with oblique Geomin rotation. The optimal number of factors to be retained was determined using the Parallel Analysis Method [63] with 5000 resamples. The eigenvalues of the sample correlation matrix were compared with the eigenvalues obtained from a randomly generated data set. Only factors with eigenvalues above the 95^th^ percentile of those generated were retained. Eigenvalues above 1.0, the percentage of explained total variance, and the overall model fit of each factor solution were also examined. Factors with less than 2 items were not considered. Items were retained when they had factor loadings ≥ .40 on the target factor and cross-loadings < .32 [64]. Subsequently, to cross-validate the EFA solution, a CFA was performed with the second half-split sample. Lastly, internal consistency was examined in the whole sample using Cronbach’s alpha.

In a third stage, multigroup CFA was conducted to test the measurement invariance of the resulting solution across gender (boys and girls) and three age groups (13–15 years old, 15–18 years old, 19–25 years old). As recommended by Meredith [65], a series of increasingly restrictive nested models were performed: configural invariance (i.e., the pattern of factor loadings is identical for both groups but no equality constrains are imposed), metric invariance (i.e., equal factor loadings across groups), and scalar invariance (i.e., equal item intercepts and factor loadings across groups). Invariance assumptions held if the fit of each highly constrained nested model was not significantly worse than the fit of the previous model. As indicators of model invariance, the ΔCFI should be below the cut-off of .01 [66].

To obtain validity evidence based on relationships between the dimensions of the SMU-SNS scale and other variables, bivariate correlations were computed. Additionally, gender and age differences in the motives for use of SNS were tested using Univariate Analyses of Variance (ANOVA).

Model fit was evaluated using a combination of indices, including the ratio of chi-square/degrees of freedom (*χ*^2^/df values around 2 or lower indicate good fit), the Comparative Fit Index and the Tucker Lewis index (CFI/TLI ≥ .90 for reasonable fit and CFI/TLI ≥ .95 for good fit), the Root Mean Square of Residuals (SRMR < .09), and the Root Mean Square of Approximation (RMSEA < .06 for adequate fit) [67].

The correlations, EFA, CFA, and multi-group analyses were conducted in Mplus7.4 using Maximum Likelihood Estimation with Robust Standard Error (MLR) to correct for potential non-normality in the variables [68]. Descriptive statistics and Cronbach’s alphas were performed with SPSS25. Missing data were treated using pairwise deletion in SPSS and Full Information Maximun Likelihood (FILM) in Mplus.

## Results

### Item-level analyses of the SMU-SNS

Item scores ranged from 1 to 7 and standard deviations exceeded 1.00, indicating adequate variability. The means were between 2.02 and 5.63. Statistics for skewness (ranging from -1.21 to 1.41, *SE* = 08) and kurtosis (ranging from -1.30 to 1.49, *SE* = .15) fell within the range of acceptable values (<|±3| and < |±10|, respectively) for a normal distribution [65]. Additionally, corrected item-total correlations between the item and the dimension to which it is supposed to belong were all above .30 (*M* = .48, *SD* = .08).

### Exploratory factor analysis of the SMU-SNS (first half-split sample- *n*_1_ = 517)

The Kaiser-Meyer-Olkin (KMO) Measure of Sampling Adequacy was .88 and the Barletts’ Test of Sphericity, χ^2^(528) = 8816.84, *p* < .001, indicated data set adequacy for factoring. The parallel analysis with 5000 resamples suggested extracting 8 factors, and examination of eigenvalues revealed that 9 factors had eigenvalues above 1.00. As recommended [62], multiple factor models were run, requesting both greater and fewer factors than the number suggested by the parallel analysis. Fit indices for the solutions with seven, eight, and nine factors are displayed in Table 4.

**Table 4:** Fit indices for the EFAs with seven, eight, and nine factors.

	*χ*^2^/df	CFI	TLI	SRMR	RMSEA
Seven-factors	3.89	.87	.80	.04	.08
Eight-factors	3.48	.90	.83	.03	.07
Nine-factors	2.03	.96	.93	.02	.04

Based on adequate fit and theoretical considerations, the nine-factor solution was selected. Specifically, this decision was based on the following criteria: (a) in contrast to the nine-factor solution that adequately fit the data, the solutions with seven and eight factors showed poor fit; (b) as indicated by the chi-square difference test, the nine-factor solution led to a significant improvement in model fit as compared to the eight-factor solution, Δ*χ*^2^(25) = 346.89, *p* < .001, (c) the solution with nine factors explained a greater percentage of total variance than the solutions with eight or seven factors; and (d) the nine-factor solution was theoretically more interpretable and consistent with the initial hypothesized structure.

In this solution, the items of the Coolness and Influencer dimensions were merged into the same factor. However, the items “to share pictures and get likes”, “to be an influencer”, and “to promote or spread my lifestyle” showed high cross-loadings (> .32) and were dropped. The resulting factor was named Social Recognition.

Additionally, the items referring to Keeping in Touch with Friends (“to hear about my friends”, “to keep in touch with friends that live far away”, and “to hear about old friends”) were discarded because of cross-loadings (> .32).

A final EFA with the remaining 27 items was subsequently conducted confirming the good fit of the nine-factor solution, *χ*^2^/df = 1.32, CFI = .99, TLI = 98, SRMR = .01, RMSEA = .03, which explained 66.89% of total variance (Table 5).

**Table 5:** EFA for the final 27-item, item descriptive statistics and factor loadings.

Factors	Items	*M* (*SD*)	Skewness (*SE*)	Kurtosis *(SE*)	λ	h
Dating (DAT) α = .90, 8.66% explained variance	To hook up	2.53 (1.67)	0.80 (0.11)	-0.53 (0.22)	0.88	.80
	To look for a date	2.17 (1.45)	1.11 (0.11)	0.22 (0.22)	0.82	.73
	To seek a romantic partner	2.05 (1.40)	1.35 (0.11)	1.06 (0.22)	0.85	.73
New Friendships (NF) α = .87, 7.97% explained variance	To make new friends	3.58 (1.53)	0.00 (0.11)	-0.75 (0.21)	0.75	.61
	To extend my circle of friends	3.82 (1.65)	-0.12 (0.11)	-0.88 (0.22)	0.72	.68
	To meet new people	4.19 (1.73)	-0.29 (0.11)	-0.73 (0.22)	0.85	.75
Academic Purposes (ACAD) α = .88, 7.55% explained variance	To ask for information about what to study for the exams	3.43 (2.01)	0.26 (0.11)	-1.25 (0.22)	0.81	.70
	To ask or share class notes	3.39 (1.95)	0.23 (0.11)	-1.25 (0.22)	0.91	.83
	To check or share group assignments	3.12 (1.74)	0.39 (0.11)	-0.85 (0.22)	0.73	.65
Social Connectedness (SC) α = .83, 7.44% explained variance	To not feel disengaged from the world	3.59 (1.83)	0.09 (0.11)	-1.12 (0.21)	0.85	.66
	To feel connected with people	4.28 (1.67)	-0.42 (0.11)	-0.73 (0.22)	0.69	.67
	To feel socially integrated	3.50 (1.74)	0.10 (0.11)	-0.92 (0.22)	0.71	.66
Following and Monitoring Others (FMO) α = .86, 7.44% explained variance	To keep up-to-date with what my contacts are doing in their day-to-day life	4.40 (1.74)	-0.37 (0.11)	-0.78 (0.21)	0.97	.86
	To know the details of my friends´ lives	4.12 (1.69)	-0.28 (0.11)	-0.73 (0.21)	0.66	.69
	To snoop on people that I am interested in	4.04 (1.93)	-0.16 (0.11)	-1.15 (0.22)	0.66	.53
Entertainment (ENT) α = .75, 7.37% explained variance	To fill my free time	4.89 (1.43)	-0.66 (0.11)	0.16 (0.22)	0.69	.55
	To kill time when I am bored	5.58 (1.24)	-0.97 (0.11)	1.23 (0.22)	0.88	.78
	To entertain myself	5.55 (1.16)	-1.04 (0.11)	1.93 (0.22)	0.74	.67
Social Recognition (SR) α = .80, 7.27% explained variance	To stand out from others	2.45 (1.42)	0.93 (0.11)	0.31 (0.22)	0.68	.53
	For other people to comment my posts	2.95 (1.71)	0.56 (0.11)	-0.68 (0.22)	0.97	.90
	To check that others like my posts	3.80 (1.86)	-0.04 (0.11)	-1.05 (0.22)	0.60	.58
Self-expression (SELF) α = .82, 6.47% explained variance	To express my feelings and thoughts	3.64 (1.43)	0.06 (0.11)	-0.63 (0.21)	0.66	.55
	To give my opinion on a topic	3.85 (1.59)	-0.09 (0.11)	-0.69 (0.22)	0.65	.60
	To discuss some subject (with other people)	3.36 (1.70)	0.30 (0.11)	-0.80 (0.21)	0.83	.68
Information (INFO) α = .77, 6.29% explained variance	To keep up about what happens in the world	5.22 (1.36)	-0.76 (0.11)	0.34 (0.22)	0.71	.58
	To be informed about the news	4.94 (1.53)	-0.71 (0.11)	0.02 (0.22)	0.85	.71
	To find information about the topics that I like and am interested in	5.53 (1.34)	-1.03 (0.11)	1.04 (0.22)	0.57	.38

Note: *M* = Mean, *SD* = Standard Deviation, *SE* = Standard Error, λ = Factor loading of the Geomin rotated solution, *h* = communality.

Thus, the final form of the SMU-SNS had 27 items on nine factors. “Thus, the final form of the SMU-SNS had 27 items on nine factors (see English version at S1 Table; the original Spanish version could be accessed at https://doi.org/10.6084/m9.figshare.9956480).

These dimensions along with their definitions were:

- Dating (DAT): Motives related to finding a romantic partner, dating or having sexual encounters.
- New Friendships (NF): Motives related to the expansion of the offline network: making new friends and meeting new people.
- Academic Purposes (ACAD): Motives related to obtaining information and help on academic matters such as exams, class notes, or group assignments.
- Social Connectedness (SC): Motives related to feeling part of society through online social networks.
- Following and Monitoring Others (FMO): Motives related to following and looking for details of the lives of ones´ friends through their profiles and publications.
- Entertainment (ENT): Motives related to using SNS for fun and passing time.
- Social Recognition (SR): Motives related to looking for popularity and feedback (likes and comments).
- Self-expression (SELF): Motives related to the expression of one's opinions and feelings.
- Information (INFO): Motives related to being aware of news and current social issues.

Except for the factor Social Recognition that included some items referring to the initial dimensions of Coolness and Influencer, the item distribution in each factor coincided with the initial proposed structure. The correlations between the factors are displayed in Table 6.

**Table 6:** Descriptive statistics and correlations among the SMU-SNS factors in the EFA.

Factors	*M*	*SD*	1	2	3	4	5	6	7	8	9
1. DAT	2.24	1.37	1								
2. NF	3.88	1.48	.45***	1							
3. ACAD	3.36	1.72	.25***	.24**	1						
4. SC	3.83	1.51	.23***	.40***	.22***	1					
5. FMO	4.21	1.56	.16***	.25***	.16***	.49***	1				
6. ENT	5.36	1.07	.10*	.25***	.17***	.40***	.45***	1			
7. SR	3.10	1.40	.33***	.38***	.17***	.50***	.50***	.32***	1		
8. SELF	3.68	1.39	.19***	.35***	.36***	.26***	.21***	.27***	.26***	1	
9. INFO	5.23	1.20	-.05	.08	.23***	.21***	.28***	.29***	.12***	.34***	1

Note: DAT = Dating, NF = New Friendships, ACAD = Academic Purposes, SC = Social Connectedness, FMO = Following and Monitoring Others, ENT = Entertainment, SR = Social Recognition, SELF = Self-expression, INFO = Information. Means and Standard Deviations are based on individuals with full information available on these variables.

* *p* < .05

** *p* < .01

*** *p* < .001.

### Confirmatory factor analysis of the SMU-SNS (second half-split sample 2 –*n*_2_ = 522)

Based on the EFA results, a nine-factor model for the final 27-item SMU-SNS was cross-validated on the second half-split sample (*n*_2_ = 522). To evaluate the goodness of fit of the resulting model, a CFA was conducted. This model fitted the data well, *χ*^2^/df = 2.42, CFI = .93, TLI = .92, SRMR = .04, RMSEA = .05. Standardized factor loadings ranged from .53 to .90 (*M* = .78) and were all statistically significant.

### Reliability of the set of indicators of the SMU-SNS

Internal consistency for all the factors was satisfactory, with Cronbach’s alpha values ranging between .77 and .90 (Table 5).

### Measurement invariance by gender and age of the SMU-SNS

Before invariance testing, model fit for the nine-factor solution was examined separately for each gender (boys and girls) and age (13–15 years old, 16–18 years old, and 19–25 years old) groups. The model fit data reasonably well for boys and girls and for the different age-groups (see Table 7), thereby providing support for the nine-factor solution as the best fitting model.

**Table 7:** Invariance tests of the SMU-SNS across gender and age groups.

	Model fit					Model difference	
	χ^2^/*df*	CFI	TLI	SRMR	RMSEA	Model comparison	ΔCFI
***Gender invariance***							
Single-group solutions							
· Boys (*n* = 455)	1.90	.95	.94	.04	.05		
· Girls (*n* = 584)	2.50	.94	.92	.05	.05		
Invariance models							
1. Configural	2.20	.941	.93	.05	.05	-	-
2. Metric	2.22	.939	.93	.05	.05	2 vs. 1	-.002
3. Scalar	2.34	.929	.92	.05	.05	3 vs. 2	-.01
3.1. Partial scalar	2.25	.932	.93	.05	.05	3.1 vs. 2	-.007
***Age invariance***							
Single-group solutions							
· 13–15 (n = 353)	1.83	.96	.92	.05	.05		
· 16–18 (n = 449)	1.99	.95	.94	.04	.05		
· 19–25 (n = 237)	1.77	.93	.91	.05	.06		
Invariance models							
1. Configural	1.87	.939	.93	.05	.05	-	
2. Metric	1.81	.941	.93	.05	.05	2 vs. 1	.002
3. Scalar	1.83	.938	.93	.05	.05	3 vs. 2	-.003

Next, invariance tests were performed for gender and age (see Table 7). For gender invariance, the adequate fit of the configural model suggested that the nine-factor solution adequately represented the data for boys and girls. Additionally, the model constrained at the metric level (i.e., factor loadings) yielded adequate fit that was not significantly worse than the configural model. The scalar constrained model (i.e., item intercepts) also fit the data well, yet it resulted in a decrement in overall model fit. Based on the modification indices, we found that the estimated intercept values for the items “to express my feelings and thoughts” (2.48 for boys and 2.61 for girls) and “to check that others like my posts” (1.99 for boys and 2.24 for girls), were operating differentially across boys and girls. Subsequently, partial scalar invariance was explored by relaxing the constraints of these intercepts across groups. The re-specified model fit the data well and according to the negligible change in the CFI, produced a similar fit as compared with the metric model. Thus, these results indicated that the SMU-SNS showed invariant factor loadings, as well as, largely invariant intercepts across gender groups.

For age invariance, all constraints at the metric (i.e., factor loadings) and scalar (i.e., item intercepts) levels were found to hold across groups as none of the CFI changes were close to the critical value of .01. Accordingly, results provided evidence of full measurement invariance across age groups.

### Validity evidence based on the relationships of the SMU-SNS with other variables

Validity evidence based on relations to other variables was obtained through a series of correlational analyses between the SMU-SNS factors and the global scores of extraversion, neuroticism, peer social support, loneliness, and life-satisfaction (see Table 8).

**Table 8:** Correlations between SMU-SNS factors and other relevant variables.

	BFI-EX	BFI-NE	MSPSS	UCLA	SWLS
DAT	.17***	-.14***	-.01	.02	.01
NF	.15***	-.08*	.09**	.06	.02
ACAD	.09*	-.02	.07*	-.04	.08*
SC	.10*	.06	.05	.15***	.02
FMO	.11**	.19***	.14**	.04	.12***
ENT	.07*	.09**	.13**	.03	.06
SR	.22***	.05	.05	.02	.09**
SELF	.12***	.00	.13***	.08*	.04
INFO	.00	.02	.13***	-.03	.15***

Note: DAT = Dating, NF = New Friendships, ACAD = Academic Purposes, SC = Social Connectedness, FMO = Following and Monitoring Others, ENT = Entertainment, SR = Social Recognition, SELF = Self-expression, INFO = Information, BFI-EX = BFI-extraversion, BFI-NE = BFI-neuroticism, MSPSS = Peers´ social support, UCLA = Loneliness, SWLS = Life satisfaction.

* *p* < .05

** *p* < .01

*** *p* < .001.

### Gender and age differences in the motives for SNS use

To explore adolescents and youths’ motives for using SNS, descriptive statistics (Means and Standard Deviations) of the SMU-SNS factors were calculated. As previously shown in Table 6, Entertainment, Information, and Following and Monitoring Others were the strongest motives for SNS use, followed by motives of New Friendships, Social Connectedness, Self-expression, Academic Purposes, and Social Recognition. In contrast, Dating has the lowest mean score.

Later, several ANOVAs were conducted to investigate gender differences in the motives for using SNS. Results revealed that boys were more likely to use SNS for Dating [*M*boys = 2.68 vs. *M*girls = 1.89, *F*(1, 1027) = 94.65, *p* < .001, partial*η*^*2*^ = .08] and New Friendships [*M*boys = 3.98 vs. *M*girls = 3.79, *F*(1, 1027) = 3.83, *p* = .049, partial*η*^2^ = .01]. Whereas girls were more inclined than boys to use SNS for Social Connectedness [*M*boys = 3.63 vs. *M*girls = 4.00, *F*(1, 1027) = 14.21, *p* < .001, partial*η*^2^ = .01], Following and Monitoring Others [*M*boys = 3.70 vs. *M*girls = 4.61, *F*(1, 1027) = 94.43, *p* < .001, partial*η*^2^ = .08], Entertainment [*M*boys = 5.24 vs. *M*girls = 5.45, *F*(1, 1027) = 9.13, *p* = .003, partial*η*^2^ = .01], and Information [*M*boys = 5.05 vs. *M*girls = 5.37, *F*(1, 1027) = 17.82, *p* < .001, partial*η*^2^ = .02]. No other significant differences were found.

Finally, ANOVAs also showed significant age differences in some of the motives for using SNS. Results indicated that participants older than 18 years (*M* = 1.98) were less likely to use SNS for Dating [*F* (2, 1026) = 5,57, *p* = .004, partial*η* ^2^ = .01] compared to the participants aged 13–15 (*M* = 2.29) or 16–18 (*M* = 2.33), as it was indicated in post-hoc analysis. A similar result was found regarding New Friendships [*F* (2, 1026) = 10,73, *p* < .001, partial*η* ^2^ = .02], with participants over 18 years old (*M* = 3.51) scoring below those of 16–18 (*M* = 3.91) or 13–15 years old (*M* = 4.07). In contrast, participants over 18 (*M* = 5.58) scored higher on Information motive [*F* (2, 1026) = 19,25, *p* < .001, partial*η* ^2^ = .04], in comparison to those of 16–18 years old (*M* = 5.23), which, in turn, were above those of 13–15 years old (*M* = 4.97). Regarding Academic Purposes, both the 16–18 years old (*M* = 3.54) and the ones above 18 (*M* = 3.51), scored higher than those with 13–15 years [*F* (2, 1026) = 10,12, *p* < .001, partial*η* ^2^ = .02]. No other significant differences were found.

### Correlations between motives for SNS use and some psychosocial variables

In order to obtain externally validated evidences, correlations were calculated between motives for SNS use and some psychosocial variables we considered relevant from the literature review. The BFI-extraversion score showed a pattern of positive correlations with all the motives for SNS use, except for Information. However, correlations were significant with the dimensions of Dating, New Friendships, and Social Recognition. The BFI-neuroticism showed a negative correlation with Dating and Following and Monitoring Others. Peer social support correlated positively with the SMU-SNS dimensions of New Friendships, Academic Purposes, Entertainment, Following and Monitoring Others, Self-expression, and Information. Loneliness was positively correlated with Social Connectedness and Self-expression. Finally, life satisfaction displayed a positive correlation with Following and Monitoring Others, Social Recognition, Information, and Academic Purposes.

## Discussion

Having reviewed the prior research on the motives for using SNS based on the Uses and Gratifications theory, we arrive at the same conclusion as that expressed by other authors such as Orchard et al. [28]: those studies did not guarantee to examine all possibly relevant motives for using SNS. Therefore, the aim of this current study was to develop and validate a scale that would allow a wide and significant range of adolescent and youth’s motives for using SNS to be evaluated.

The results of the SMU-SNS scale cross-validation using EFA and CFA supported a 9-factor first-order model comprised of the motives Dating, New Friendships, Academic Purposes, Social Connectedness, Following and Monitoring Others, Entertainment, Social Recognition, Self-expression, and seeking Information. All dimensions showed adequate internal consistency. In addition, the internal structure of the SMU-SNS demonstrated to be invariant across age. However, some differences were found in the case of measurement invariance across gender. The same pattern of factor loadings was found for boys and girls (metric invariance), yet only partial scalar invariance was supported since two item-intercepts for the Social Recognition and Self-expression factors were larger in girls than in boys, thereby suggesting that girls scored systematically higher than boys on both items.

The SMU-SNS includes some dimensions to measure motives for using SNS common to other studies (see Table 2 in Section 1.1). However, the novelty and strength of the SMU-SNS lies in the fact that it allows a broad range of motives to be evaluated using a single instrument and a rigorous methodological process for its development. This process included a literature review, an analysis of the validated instruments already available and, finally, the results of a mixed study conducted to select the relevant and significant motives of use among adolescents and youths.

The study has also offered externally validated evidence that emphasizes the need to consider the motives for using SNS when studying the relationship between use and certain psychological variables that may be either a precursor and/or consequence of use. In general terms, the results indicate that certain profile characteristics of users (gender, age, and personality) interact differently with their motives and needs. Gender is a key variable when characterizing and understanding SNS users on an individual level. However, authors such as Trauth [69] believe that more empirical evidence is needed in order to fully understand how gender influence the patterns of SNS use.

Nonetheless, our research has corroborated some gender differences in the motives of use which prior literature has already partially demonstrated. Specifically, girls tend to use SNS more for following pre-existing friends as a form of maintaining contact, whereas boys report more use for establishing new friendships and romantic relationships. We believe that these differences can be interpreted based on the Self-Construal Theory of Gender [14,70]. According to this theory, the initial tendency of girls towards a relational self-construal would indicate more interest in maintaining intimate and exclusive relationships, which in the case of SNS would relate with behavior motivated by monitoring your closest network of contacts. On the other hand, the tendency attributed to boys of collective self-construal would mean that they give more importance to building a wide network of friends, which in the case of SNS would be reflected in behaviors motivated by establishing new friendships and romantic relationships. Ultimately, it seems that there are gender differences both in online and offline life regarding the relational motives, explained by the relational and collective self-construal, respectively.

Secondly, although there has been little evidence on age-related motives for use, the scale has allowed us to examine some developmental tendencies. For example, younger users report more frequent usage of SNS to establish new contacts, whereas older users are more driven by motives related to following and maintaining contact with pre-existing friends. It is assumed that as one ages, youth tend to establish a more stable social circle. In addition, the increased maturity comes with a decrease in motivation to establishing new relationships. In this sense, Orchard et al. [28] found that age not only predicted higher conformity but also a lower motivation to establish new contacts online. Finally, in a similar way we found that older participants used SNS for motives associated to seek information and for academic purposes, which can be interpreted as increased maturity too, with an expected higher social and academic commitment at that age.

On the other hand, further research is needed to clarify how the users’ personality difference could associate with different motives, uses, and specific behaviors online [28]. Extraverts tended to use SNS more actively, which involves extensively using the different functions and possible means of communication that they offer. In our study, extraversion significantly correlated with social motives, such as seeking social recognition, as well as establishing new friendships and dating. These findings are consistent with the nature of extraverts and with the “rich get richer” hypothesis [71]. Extraverts have a need to expand their social network, which is why they would use SNS as a means to widen their circle and therefore have more online contacts, as has been consistently demonstrated. On the contrary, introverts have smaller online social networks, do not like social recognition and prefer other more anonymous virtual environments in which to express themselves and relate to others [28].

Regarding neuroticism, we found an expected positive association with the dimension of following and monitoring others and a negative association with dating. It should be noted that neuroticism has been frequently associated with spending more time on SNS [72]. Therefore, the most neurotic subjects tend to use SNS passively and the time dedicated to them supposes a type of pastime consistent with following others online without communicating nor interacting much with other people [28,39]. Therefore, higher neuroticism relates with a higher control over information shared online, showing a lower tendency to communicate with a wide audience for fear of not being accepted by others, and a high difficulty for using private or direct messages online, which is usually the common means of establishing contact with people that one finds interesting or likes [34,35].

Lastly, it is necessary to clarify how age, gender, and personality can associate with the use of SNS and if this use could have a positive influence on adolescent and youth’s wellbeing. In our study, we have found a positive association of the dimensions online self-expression and following and monitoring others with a higher perception of social support. On the other hand, loneliness related to seeking social connection through SNS, probably as social compensation, which was related to isolation in real life. Lastly, higher life satisfaction was associated with following others through SNS and with the need to be socially informed about your group of contacts and friends. Therefore, a possible positive use of SNS can be deduced from these findings. That is, if youths use SNS as another way to maintain contact with real-life friends, this use could be related with a higher perception of social support, lower perception of loneliness, and consequently, greater subjective wellbeing [73].

### Limitations and implications for future research

The limitations of the study will now be addressed. Some limitations are inherent to the study and the sample. One must consider that the motives for use may change between types of SNS according to their nature and the activities that they allow and encourage. Therefore, in order to better contextualize and understand the results of our study we must specify that the majority of our sample reported that Instagram was their favorite and most frequently used SNS. Currently, this tendency of use, at least in the Spanish context, is predominant amongst adolescents and youths (whereas Facebook is the most widely used SNS in adults). Therefore, these results should be interpreted cautiously and need to be validated in adolescent and youth populations in other countries according to the most used or current SNS in those contexts.

Secondly, although our sample covers a wide range of ages between adolescence and young adulthood, this study should be replicated in an adult population in order to obtain evidences of the validity of the SMU-SNS in this age group in our context.

A third limitation is the need for more externally validated evidences. Thus, other variables related psychological adjustment would have ideally been used in order to examine the relationship with patterns and motives of use of SNS. Likewise, one must keep in mind that the data are transversal and do not allow casual relationships to be established. Future research should study whether certain motives of use predict an increase or decrease of subjective wellbeing, which is why it would be interesting to conduct longitudinal studies that determine the likely causal relationship between motives of use and psychological variables.

Likewise, the Spanish version of our instrument would need to be adapted and validated for use in Latin-American countries (a version in Spanish can be provided upon request), as well as an English version for other cultural contexts, in order to verify if the dimensions are also significant and relevant and if they behave similarly to what was found in our study.

In conclusion, we believe it was necessary to develop and validate an instrument to explore in a more comprehensive way the motives of SNS use amongst adolescents and youths. The relevance of this study lies in going beyond merely examining the different patterns of online use, but rather to be able to demonstrate, consistent with prior literature, how the different motives of use can potentially mediate the relationship between adolescent and youth’s use of SNS and their psychological wellbeing in an ample sense. In this respect, future research should explore the predictive value of the uses in relationship to wellbeing.

Finally, it is necessary to note the Uses and Gratifications Theory, which proposes a narrow-focused approach to explain the motives for using SNS. However, to deeply understand the motives and psychological experiences derived from using SNS it seems important to mention an approach with a broader focus such as the Self-Determination Theory and the Basic Needs Theory [74]. In order to gain further insight into the use of SNS we consider it interesting to explore their relationship to the overall satisfaction of the three basic psychological needs for competence, relatedness, and autonomy, and how these are experienced and connect between online and offline life. For their inherently social nature, SNS appear to be clearly designed to satisfy the need to relate with others in a virtual context. Online communication might either foster need satisfaction as well as compensate face-to-face relationships when this psychological need is frustrated, or cause more need frustration and increase isolation [75]. Future research can enlighten how SNS use might be related to the satisfaction or frustration of basic psychological needs.

## Supporting information

S1 File Data set of the study. (DAT)

S1 Table Items of the Scale of Motives for Using Social Networking Sites (SMU-SNS) for adolescents and youths–English version. (DOCX)
