# Supplementary material for: Development and validation of the Scale of Motives for Using Social Networking Sites (SMU-SNS) for adolescents and youths
Source: PLoS One. 2019 Dec 3;14(12):e0225781. doi: 10.1371/journal.pone.0225781 (PMC6890241; doi:10.1371/journal.pone.0225781)
Supplement: S1 Table — (DOCX) [file pone.0225781.s002.docx]

**S1 Table. Items of the Scale of Motives for Using Social Networking Sites (SMU-SNS) for adolescents and youths –English version.**

The following statements are about the motives for using SNS. Read them and answer indicating to which extent they apply to you.

| **1** | **2** | **3** | **4** | **5** | **6** | **7** |
| --- | --- | --- | --- | --- | --- | --- |
| **Completely untrue** |  |  |  |  |  | **Completely true** |

| 1. | To hook up | **1** | **2** | **3** | **4** | **5** | **6** | **7** |
| --- | --- | --- | --- | --- | --- | --- | --- | --- |
| 2. | To look for a date | **1** | **2** | **3** | **4** | **5** | **6** | **7** |
| 3. | To seek a romantic partner | **1** | **2** | **3** | **4** | **5** | **6** | **7** |
| 4. | To make new friends | **1** | **2** | **3** | **4** | **5** | **6** | **7** |
| 5. | To extend my circle of friends | **1** | **2** | **3** | **4** | **5** | **6** | **7** |
| 6. | To meet new people | **1** | **2** | **3** | **4** | **5** | **6** | **7** |
| 7. | To ask for information about what to study for the exams | **1** | **2** | **3** | **4** | **5** | **6** | **7** |
| 8. | To ask or share class notes | **1** | **2** | **3** | **4** | **5** | **6** | **7** |
| 9. | To check or share group assignments | **1** | **2** | **3** | **4** | **5** | **6** | **7** |
| 10. | To not feel disengaged from the world | **1** | **2** | **3** | **4** | **5** | **6** | **7** |
| 11. | To feel connected with people | **1** | **2** | **3** | **4** | **5** | **6** | **7** |
| 12. | To feel socially integrated | **1** | **2** | **3** | **4** | **5** | **6** | **7** |
| 13. | To keep up-to-date with what my contacts are doing in their day-to-day life | **1** | **2** | **3** | **4** | **5** | **6** | **7** |
| 14. | To know the details of my friends’ lives | **1** | **2** | **3** | **4** | **5** | **6** | **7** |
| 15. | To snoop on people that I am interested in | **1** | **2** | **3** | **4** | **5** | **6** | **7** |
| 16. | To fill my free time | **1** | **2** | **3** | **4** | **5** | **6** | **7** |
| 17. | To kill time when I am bored | **1** | **2** | **3** | **4** | **5** | **6** | **7** |
| 18. | To entertain myself | **1** | **2** | **3** | **4** | **5** | **6** | **7** |
| 19. | To stand out from others | **1** | **2** | **3** | **4** | **5** | **6** | **7** |
| 20. | For other people to comment my posts | **1** | **2** | **3** | **4** | **5** | **6** | **7** |
| 21. | To check that other like my posts | **1** | **2** | **3** | **4** | **5** | **6** | **7** |
| 22. | To express my feelings and thoughts | **1** | **2** | **3** | **4** | **5** | **6** | **7** |
| 23. | To give my opinion on a topic | **1** | **2** | **3** | **4** | **5** | **6** | **7** |
| 24. | To discuss some subject (with other people) | **1** | **2** | **3** | **4** | **5** | **6** | **7** |
| 25. | To keep up about what happens in the world | **1** | **2** | **3** | **4** | **5** | **6** | **7** |
| 26. | To be informed about the news | **1** | **2** | **3** | **4** | **5** | **6** | **7** |
| 27. | To find information about the topics that I like and am interested in | **1** | **2** | **3** | **4** | **5** | **6** | **7** |
